# The Open Basement

**Published:** 1914-02-21

**Authors:** G. A. Bligh Livesay

**Affiliations:** Ardmore, Parkstone, Dorset


### THE EDITOR'S LETTER-BOX.

### The Open Basement.

*To the Editor of* The Hospital.

Sir,—I thank you for your excellent reference in Saturday's issue of The Hospital. I am very glad you brought out the point about the *rubbish* under the wards (from which I have often suffered!), though recently cleared by the Secretary's orders. So much for the *abuse*; now for the remedy!

The remedy is simple, though it is hard indeed to get committees to realise that loose paper, dogs, and cats (let alone garden seats, tables, chairs, and the like articles) can only be kept out by filling in the arches or openings permanently with galvanised wire netting or bars in frames, leaving access *only* by means of one or two of the grids being hung to open and provided with padlocks, the keys being in charge of the matron or lady superintendent. Moreover, the ground surface under the wards should be covered with asphalt paving on rolled gravel, which the gardener would have orders to hose down every few months.

An added advantage of the open basement, not touched on in my paper, is that all heating and water service pipes are hung on stout hangers or clips under the ward floors, and in case of a burst pipe or leak the shut-off valves for the various circuits are easily accessible at a man's height, as also are the pipes themselves for any repairs that may be necessary.

Being in the open air all pipes are lagged to retain heat, and the expense of laying pipes in specially constructed trenches is avoided.—Yours faithfully,

G. A. Bligh Livesay, F.R.I.B.A.

Ardmore, Parkstone, Dorset.

